# *Bacillus anthracis* Protease InhA Increases Blood-Brain Barrier Permeability and Contributes to Cerebral Hemorrhages

**DOI:** 10.1371/journal.pone.0017921

**Published:** 2011-03-17

**Authors:** Dhritiman V. Mukherjee, Jessica H. Tonry, Kwang Sik Kim, Nalini Ramarao, Taissia G. Popova, Charles Bailey, Serguei Popov, Myung-Chul Chung

**Affiliations:** 1 National Center for Biodefense and Infectious Diseases, Department of Molecular and Microbiology, George Mason University, Manassas, Virginia, United States of America; 2 Division of Pediatric Infectious Diseases, Johns Hopkins University School of Medicine, Baltimore, Maryland, United States of America; 3 Unité MICALIS/GME, INRA, La Minière, Guyancourt, France; Cairo University, Egypt

## Abstract

Hemorrhagic meningitis is a fatal complication of anthrax, but its pathogenesis remains poorly understood. The present study examined the role of *B. anthracis*-secreted metalloprotease InhA on monolayer integrity and permeability of human brain microvasculature endothelial cells (HBMECs) which constitute the blood-brain barrier (BBB). Treatment of HBMECs with purified InhA resulted in a time-dependent decrease in trans-endothelial electrical resistance (TEER) accompanied by zonula occluden-1 (ZO-1) degradation. An InhA-expressing *B. subtilis* exhibited increased permeability of HBMECs, which did not occur with the isogenic *inhA* deletion mutant (*ΔinhA*) of *B. anthracis*, compared with the corresponding wild-type strain. Mice intravenously administered with purified InhA or nanoparticles-conjugated to InhA demonstrated a time-dependent Evans Blue dye extravasation, leptomeningeal thickening, leukocyte infiltration, and brain parenchymal distribution of InhA indicating BBB leakage and cerebral hemorrhage. Mice challenged with vegetative bacteria of the *ΔinhA* strain of *B. anthracis* exhibited a significant decrease in leptomeningeal thickening compared to the wildtype strain. Cumulatively, these findings indicate that InhA contributes to BBB disruption associated with anthrax meningitis through proteolytic attack on the endothelial tight junctional protein zonula occluden (ZO)-1.

## Introduction

*Bacillus anthracis* is a gram-positive, spore-forming, rod-shaped bacterium that causes three distinct clinical forms of anthrax depending on the major routes of infection: inhalational, cutaneous, and gastrointestinal [1]. All forms of anthrax can be highly lethal if left untreated, due to systemic spread of bacteria through hematogenous and lymphatic routes. At the systemic stage, anthrax is accompanied by damage to many organs and tissues. Clinical case reports indicate that bacteria disseminated to the brain can cause hemorrhagic meningitis, a life-threatening pathology manifested as a “cardinal's cap”, due to the characteristic appearance of meninges upon autopsy [1]–[4]. During the epidemic of inhalation anthrax in Sverdlovsk, Russia, fatal hemorrhagic meningitis was reported in 50% of the individuals infected with aerosolized *B. anthracis* spores [3], [4]. Further studies revealed that 79% of all cases contained bacilli in meninges, and low-pressure meningeal hemorrhages were found in 90% of all autopsies [4]. Under physiological conditions, access of pathogens or macromolecules to the central nervous system (CNS) is restricted by the anatomic and functional unit called the blood-brain barrier (BBB) which is selectively permeable to some hormones and nutrients. Histopathological analysis indicates that during anthrax the secreted pathogenic factors of *B. anthracis* exert devastating effects on the BBB, rendering it leaky and permeable [2]–[4].

The major virulence determinants of *B. anthracis* are encoded by two large plasmids, pXO1 and pXO2. The genes for the Lethal Toxin (LT) and Edema Toxin (ET) are located on pXO1, and genes for the anti-phagocytic poly-γ-D-glutamic acid capsule are located on pXO2. LT is a specific protease inactivating mitogen-activated protein kinases in the host cells, while ET is an adenylate cyclase. Both toxins influence a broad spectrum of cellular functions, including changes in vascular permeability leading to barrier dysfunction manifested as tissue edema and pleural effusions [5]. However, the activity of LT and ET cannot fully account for several pathological findings of anthrax, such as massive hemorrhages, intensive organ and tissue damage, and other immune consequences including degradation of cytokines and immunoglobulins, as observed in mice treated with culture filtrates of *B. anthracis* [6]. The disseminated tissue damage suggests that some secreted *B. anthracis* protease(s) distinct from LT may also serve a pathogenic role. Moreover, several bacterial proteases from species other than *B. anthracis* have been known to cause massive internal hemorrhage and life-threatening pathologies [7]–[10]. We recently purified from culture supernatant of *B. anthracis* two zinc metalloproteases: Neutral Protease 599 (Npr599 or NprB), a thermolysin-like protease, and Immune Inhibitor A (InhA), a homolog of *B. thuringiensis* protease [11]. The ability of these proteases to degrade plasma and matrix proteins *in vitro* suggests their role in endothelial barrier permeability and blood homeostatic imbalance during the infectious process [11]–[13].

The structural and functional integrity of the BBB primarily depends on the state of brain capillaries and its polarized microvascular endothelial cells which possess tight junction (TJ) [14]. Intercellular TJ proteins include occludin, claudin, junctional adhesion molecules (JAMs), and membrane-associated guanylate kinase-like proteins (MAGUK) or zonula occludens (ZO)-1 [15]. Their function is to regulate paracellular passage of substrates across the BBB. ZO-1 is associated with TJ proteins and the actin cytoskeleton, and is essential for the stability, organization and signaling of TJ proteins; loss or dissociation of the protein from its counterparts has been implicated in increasing barrier permeability [16]–[19]. It has been recently shown that proteolytic breakdown of the microvascular endothelial cell monolayers by *B. anthracis* is associated with degradation of ZO-1, a process which requires adhesion of bacteria to endothelium through the S-layer adhesin BslA [20]. Some bacterial proteases can unravel junctional complexes in endothelial and epithelial cells, thereby inducing barrier permeability in non-CNS tissues [21].

In the present study, we tested whether InhA plays a role in BBB permeability associated with anthrax meningitis. Using HBMECs as an *in vitro* model of BBB we demonstrate that increased permeability takes place upon internalization of InhA into HBMECs followed by degradation of ZO-1. Experiments with mice challenged with purified InhA or vegetative bacteria of the *ΔinhA* strain of *B. anthracis* indicate that expression of InhA contributes to hemorrhagic brain damage associated with fatal meningitis.

## Materials and Methods

### Protease isolation and purification

Npr599 and InhA were isolated from culture supernatant of *B. anthracis* Sterne 34F2 (devoid of both pXO1 and pXO2) and purified as described previously [11]. Protease gelatin hydrolytic activity was determined using the EnzChek gelatinase/collagenase kit (Molecular Probes) according to manufacturer's protocol.

### Real-time TEER measurements

Frozen stocks of HBMECs between passages 8 and 24 were thawed and then maintained as previously described [22] in RPMI 1640-based medium with growth factors, 10% fetal bovine serum (FBS), of endothelial cell growth supplement (30 *µ*g/ml), heparin (5 U/ml), L-glutamine (2 mM), sodium pyruvate (1 mM), non-essential amino acids, vitamins, penicillin and streptomycin (100 U/ml). Cultures were incubated at 37°C in a humid atmosphere of 5% CO_2_ and split in a ratio of 1∶3 twice a week.

During experiments, the original RPMI1640-based culture medium was replaced with the medium (XM) containing 1∶1 ratio of Medium 199 and Ham's F-12 medium, 5% heat-inactivated FBS, and 1 mM glutamine [22]. To estimate the integrity of the HBMECs monolayers the electric cell–substrate impedance sensing (ECIS) system was used to measure and monitor TEER in HBMECs as described in [23]. Cells were grown on gold-coated electrodes (0.8 cm^2^ per well) in microelectrode 8W10E^+^ ECIS arrays (Applied BioPhysics, NY). The voltage between the small electrode and the large counter electrode was monitored by a lock-in amplifier, stored, and processed by computer-controlled instrumentation (Applied Biophysics, NY). The electrodes/wells were coated with 50 µg/ml of rat tail collagen (BD Bioscience) prior to being washed by culture medium. HBMECs were seeded at 2×10^4^ cells/well and allowed to grow to confluence. At cell confluence, the culture medium was replaced with XM and allowed to equilibrate for 2 h within the ECIS setup prior to adding InhA and Npr599. InhA (1 µg/ml) or Npr599 (1 µg/ml) were added to the apical aspect of cells in the 8-well cell culture arrays. For bacterial infection, the recombinant *B. subtilis* 168 transformed with the InhA-expressing plasmid [24] was used as a gain-of function strain. The *ΔinhA* of *B. anthracis* Ames 35 (attenuated Ames strain lacking pXO2) generously provided by Dr. S. Leppla (National Institutes of Health) was used as a loss-of-function strain [13]. Bacteria of the mutant and corresponding isogenic parental strains were cultured overnight in LB media and then added to the HBMEC monolayers (about 6×10^4^ cells/well) at different multiplicities of infection (MOI). TEER in each well was measured at 2-min intervals for 24 h and was recorded as an average of signals from duplicate wells. Differential interference contrast microscopy was used to observed morphological changes of the exposed HBMECs cell monolayers after 24 h.

### Dextran permeability assay

Cells were seeded on collagenized transwell inserts (6.5 mm diameter, 0.4 µm pore size, Corning-Costar), grown for 7 days at 37°C in the atmosphere of 5% CO2, and then treated with the indicated amount of proteases or culture supernatants for 24 h. After incubation, culture medium in the upper chamber was replaced with Hanks Balanced Salt Solution (HBSS) containing 0.5 mg/ml of FITC-dextran (MW 40 kDa) for 2 h. Leakage of FITC-dextran to the bottom chamber was measured with a fluorescence microplate reader at 538 nm after excitation at 485 nm. Positive control included incubation with the supernatant of *B. anthracis* deltaSterne (pXO1^−^, pXO2^−^) culture grown overnight in a shaking incubator at 300 rpm in LB medium at 37°C and then diluted ten-fold with cell culture medium.

### Preparation of recombinant ZO-1 (rZO-1) protein fragment

PCR amplification of ZO-1 cDNA obtained from ATCC (Gene Bank Number: BC111712) was done using specific primers (forward 5′-TCA GAT GTA GGA GAT TCT TTC-3′ and reverse 5′-ATG CTG AGA TGA AGG TAT CAG-3′), the product was inserted into pTRcHis II TOPO (TOPO TA cloning kit, Invitrogen) and transformed into *E*. *coli* DH5-α cells.

Positive transformants were selected and verified by restriction mapping and sequencing. Expression of ZO-1 protein fragments in DH5-α cell cultures was induced by IPTG and the His_6_-tagged products were purified by Ni^2+^-NTA resin (Invitrogen). Purified recombinant proteins were incubated with InhA at molar ratio of 10∶1 in 50 mM Tris-HCl, pH 7.5, 2 mM CaCl_2_ at 37°C, and the resulting products were analyzed by Western blotting using the anti-His_6_ antibody (Invitrogen) to determine their molecular masses and the positions of InhA cleavage sites.

### ZO-1 degradation, SDS-PAGE and Western blot analysis

HBMECs (2×10^6^ cells) were treated with Npr599 (1 µg/ml) or InhA (1 µg/ml) at 37°C for different times, washed twice with HBSS, and lysed with RIPA buffer (Pierce) containing 1% Triton X-100 (Sigma) and 1x protease inhibitor cocktail (Pierce). Lysates were centrifuged at 14,000 g for 10 min and supernatants collected. InhA (1, 0.1, and 0.01 µg/ml) was also used to treat cell lysates obtained from control HBMECs as well as the rZO-1 protein at 37°C for 4 h. For Western blot analysis, samples were boiled in SDS sample buffer containing 30 mM dithiothreitol. Proteins were separated on a 4–12% Bis-Tris or 6% Tris-Glycine gels (Invitrogen) and transferred onto nitrocellulose membranes using the iBlot Gel Transfer System (Invitrogen). The membranes were blocked with 5% milk powder in PBS and probed with antibodies against ZO-1 (Invitrogen), occludin (Invitrogen), claudin-1 (Invitrogen) and JAM-1 (Chemicon). After washes, membranes were incubated with appropriate peroxidase-coupled secondary antibodies (Amersham Biosciences) and developed with Super Signal West-Femto chemiluminescent substrate (Pierce). Visualization of the blots was done with ChemiDoc XRS imager (BioRad).

### Immunostaining and fluorescence microscopy

Confluent HBMEC monolayers treated with purified InhA were washed three times with PBS, fixed with 2% paraformaldehyde in PBS for 15 min, and permeabilized with 0.5% Triton X-100 in PBS for 20 min. The cells were further incubated with the mouse anti-human ZO-1 (Zymed) or the rabbit anti-InhA antibody (custom made as described below) in PBS containing 5% fetal calf serum for 1 h, washed three times with PBS, and then incubated with the FITC-conjugated goat anti-mouse or the mouse anti-rabbit antibodies in PBS containing 5% fetal calf serum. After incubation, cells were washed with PBS, stained with DAPI ProLong Gold antifade reagent (Invitrogen), and visualized with a Nikon confocal 90i microscope.

Tissue section slides were deparaffinized, re-hydrated, fixed with 4% paraformaldehyde for 10 min, rinsed three times with PBS, and blocked with 1.5% goat serum PBS solution for 30 min at 4°C. Slides were washed again and incubated with the primary anti-InhA antibody at 4°C overnight, followed by the corresponding FITC-labeled secondary antibody as described above. The anti-InhA antibody used in these experiments was raised in rabbits against the N-terminal fragment CPAKQKAYNGDVRKD of the active form of InhA and purified from the immune serum by peptide affinity chromatography (Invitrogen).

### *In vivo* BBB permeability assays

Female, 9-week-old DBA/2 mice (Jackson Laboratory) were maintained at Biocon, Inc. (Rockville, MD). Institutional Animal Care and Use Committees at George Mason University and Biocon, Inc. approved all protocols. Mice were anesthetized by injection of 90 mg/kg of ketamine and 10 mg/kg of xylazine and then one unit of purified InhA in phosphate-buffered saline per mouse was injected *via* a retro-orbital route. One unit is defined as the amount of enzyme in 1 ml required to liberate 1 µmol of L-leucine from collagen (100 µg/ml) in 5 h at 37°C, pH 7.5. Approximately 1 h before each time point, animals were injected with a 100 µl of Evans Blue (EB) dye (1% w/v solution) through the tail vein and then perfused with 10 ml of PBS 1 h later through the apex of the heart [25]. The brains were then removed, weighed and homogenized in 1.5 ml of PBS. To extract the EB dye from the tissue homogenate, 1.5 ml of trichloroacetic acid (60% w/v) were added to each sample. The homogenate solution was vortexed for 2 min and then centrifuged for 30 min at 4°C at 1000 g. The EB supernatant was collected and absorbance measured on plate reader at 620 nm, and the background absorbance subtracted. Extravasation of dye was expressed as µg of EB dye per g of brain.

Similar to experiments with purified InhA, animals were injected with 100 µl of Quantum Dot nanoparticles (Invitrogen) conjugated with one unit of active or heat-inactivated protease. Nanoparticles-conjugated to InhA was prepared using Qdot® ITK™ carboxyl Quantum Dots according to manufacturer's recommendation (Invitrogen). Control mice were injected with equal volume of nanoparticles alone. After approximately 24 h, the animals were euthanized, the brains were removed and immersed in 10% buffered formalin for fixation. The 5-µm sections of fixed brains were stained with hematoxylin and eosin (H&E) for further histological analysis by AML Laboratories (Rosedale, MD). Immunofluorescent detection of nanoparticles containing a red fluorophore (emission at 585 nm) was performed using fluorescence microscopy. InhA was immunostained using rabbit α-InhA primary antibody and a FITC-conjugated mouse anti-rabbit antibody.

### Mouse infection studies

For a meningitis model, the *ΔinhA* of Ames 35 strain (*ΔinhA*) and the wildtype Ames 35 strain were grown overnight in luria broth. Pelleted bacteria were washed in PBS and 5×10^4^ or 5×10^5^ CFU in 0.1 ml of PBS were injected intravenously into 9-week-old female DBA/2 mice (n = 5 per each group). To determine a mean time-to-death, mice were monitored daily for 7 days. For histological analyses, mice (n = 8 per each group) were injected with 5×10^5^ CFU of vegetative bacteria and euthanized when they become moribund. Blood and brain were collected and bacterial loads were determined by CFU, serial dilutions plated on agar.

### Statistical analysis

P-values were calculated by one-way ANOVA. Statistical significance was set at *p*<0.05. Data are presented as arithmetic mean ± SD.

## Results

### Secreted *B. anthracis* metalloprotease InhA increases permeability of HBMEC monolayers

**Figure 1:**
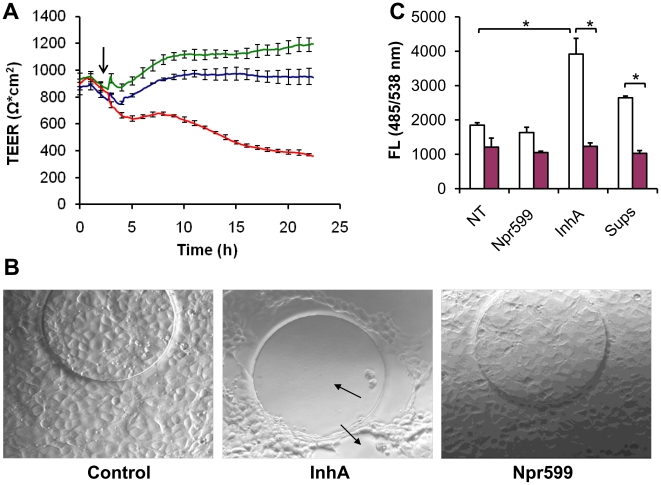
Real-time TEER of HBMEC monolayers and phase contrast microscopy. (A) Confluent HBMEC monolayers were treated at 37°C for 24 h in the atmosphere of 5% CO_2_ with 1 µg/ml of either InhA (red) or Npr599 (blue). Average traces of two wells were recorded in two-minute intervals for a 24-h period. The bars show the extent of variability between independent wells. The arrow marks the addition of protease. (B) Phase contrast microscopy of HBMEC monolayers at 24 h post treatment. The arrows point to gaps formed in the monolayers of HBMECs after treatment described in (A). InhA, center panel; Npr599, right panel; mock-treated control, left panel; 100x magnification. (C) Transwell permeability assay of HBMECs treated as in (A). Positive control included treatment with *B. anthracis* culture supernatants (Sups) diluted 10-fold with cell culture medium in the absence (white bar) or presence (red bar) of o-phenanthroline (100 µM). Leakage of FITC-dextran across monolayers was determined as described in Materials and Methods. All measurements were made in triplicates, and the experiment was repeated twice. NT, control untreated cells.

The effect of InhA on the integrity of human BBB was examined *in vitro* using HBMEC monolayers. A real-time TEER was used to measure barrier integrity and changes in confluent monolayer permeability. As shown in Figure 1A, InhA-treated HBMECs exhibited a progressive drop in TEER over time, while the mock-treated control and neutral protease (Npr599)-treated HBMECs did not exhibit a decrease in TEER. At 24 h post-exposure, TEER of the InhA-treated monolayers dropped to approximately 60% compared to the value observed in the mock-treated control. At the 24 h time point, the differential interference contrast microscopy revealed gaps in the InhA-treated HBMECs as well as the detachment of the cells from the slide surface (Figure 1B). In the case of mock- and Npr599-treated cells, the monolayers remained intact and displayed no change in morphology. HBMEC monolayers were also assayed by the transmigration of FITC-dextran (Figure 1C). Incubation of the cells with InhA and *B. anthracis* deltaSterne culture supernatant significantly increased monolayer permeability, whereas o-phenanthroline (100 µM) an inhibitor of metalloproteases abrogated the permeabilizing activity of InhA and the bacterial culture supernatant. There were no significant changes in viability of HBMECs during treatments with the proteases and culture supernatant (not shown). These data demonstrate the ability of the InhA to increase permeability of HBMEC monolayers *in vitro*.

### Cytoplasmic protein ZO-1 is a target for InhA

**Figure 2:**
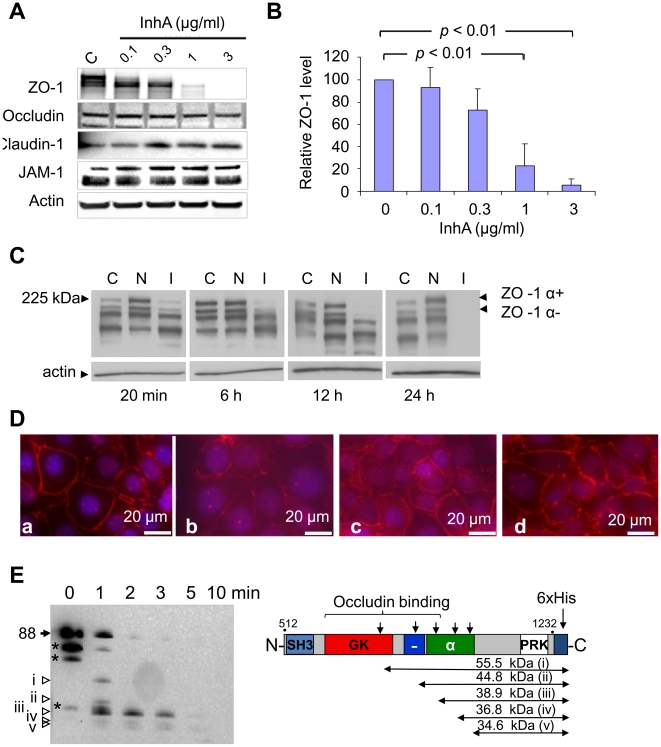
Western blot of TJ proteins in HBMECs after treatment with InhA. (A) HBMECs cells were treated with increasing concentrations of InhA (0.1, 0.3, 1, 3 µg/ml from left to right) for 24 h at 37°C. Western blots were probed with antibodies to ZO-1 (225 kDa), occludin (56 kDa), claudin-1 (22 KDa), and JAM-1 (39 kDa). (B) Densitometry of ZO-1 protein bands from (A). All samples analyzed (*n* = 4) were normalized to the intensity of corresponding β-actin bands. (C) Western blot analysis of time-dependent degradation of ZO-1 in HBMECs. Black arrows indicate two isoforms for ZO-1. *C*, control; *N*, Npr599; and *I*, InhA; α+ and α-, two splice variants of α domain. (D) Immunofluorescence of ZO-1 in HBMECs treated with or without 0.25 µg/ml of cytochalasin D for 1 h prior to incubation with varying concentrations of InhA at 37°C for 12 h. Panels show HBMECs treated as follows: untreated (a); InhA-treated (b); cytochalasin D-treated (c); InhA-treated after cytochalasin D treatment (d). Scale bar: 20 µm. (E) Western blot of His_6_-tagged rZO-1. Degradation of purified rZO-1 after treatment with 0.1 µg/ml of InhA for indicated time at 37°C. Putative cleavage sites within the rZO-1fragment after treatment with InhA were deduced from immunoblot using the molecular masses of the cleavage products. SH3, Src homology 3; GK, guanylate kinase homolog; α, 80-amino-acid splice variant; -, acidic domain; PRK, proline-rich domain. β-Actin band intensity was used as a loading control for all Western blots. The results shown in (A)-(D) are representative of 4 independent experiments.

BBB function is defined by the state of its TJ proteins and the proteins associated with them in the cell. A decrease in barrier function is directly correlated with a loss of junctional proteins [15]. We therefore examined whether InhA caused degradation of TJ proteins in HBMECs. Western blot analysis of HBMEC cell lysates after 24 h incubation with purified InhA showed degradation of ZO-1 but not claudin-1, occludin, or JAM-1 in a concentration-dependent manner (Figure 2A, B). As a control, heat-inactivated InhA did not degrade any of the above-mentioned TJ proteins (not shown). Initial signs of ZO-1 degradation by InhA were observed as early as 20 min after treatment, while a complete loss of ZO-1 was observed after 24 h (Figure 2C). Notably, Npr599 was unable to cleave ZO-1 in HBMECs; instead, it increased ZO-1 levels. Immunofluorescence microscopy confirmed the loss of ZO-1 upon treatment with InhA (Figure 2D, panel b). These results suggest that ZO-1 is a biologically relevant target of InhA in HBMECs. However, in order to access the cytoplasmic ZO-1 InhA has to be transported into the cell.

As ZO-1 is a cytoplasmic protein, we tested whether InhA could access this protein through an endocytic process. HBMECs were pre-treated with cytochalasin D, a known potent inhibitor of endocytosis. The pre-treated cells did not show any degradation or punctuation of ZO-1 upon further treatment with InhA (Figure 2D-d), indicating that degradation of ZO-1 took place after InhA entered the cell through endocytotic trafficking, a process requiring actin polymerization.

To examine whether the observed proteolytic action on ZO-1 in HBMECs can be attributed to InhA alone, or to any other intrinsic cellular proteases that might be activated in response to InhA, we generated rZO-1 protein using recombinant *E. coli* expression system. Because of difficulty in expressing a large ZO-1 molecule in *E. coli*, we chose a middle segment of ZO-1 based on our assessment of its proteolytic susceptibility to InhA (not shown). Purified rZO-1was incubated with InhA over different time periods. Western blot showed that InhA induces a time-dependent degradation of rZO-1 yielding several fragments (Figure 2E). This result demonstrates that ZO-1 is susceptible to cleavage by InhA and thus supports a possibility of direct interaction between these proteins in the cell. We were able to assign putative cleavage regions spanning from the guanylate kinase (GK) domain to the α domain, based on the molecular weight of the fragments seen in the Western blot probed with α-His_6_-tag antibody (Figure 2E). Three of the cleavage sites are located within the occludin binding domain, suggesting that InhA might be able to disrupt ZO-1's interaction with occludin.

### InhA disrupts the HBMEC monolayers *in vitro* and the BBB *in vivo*

**Figure 3:**
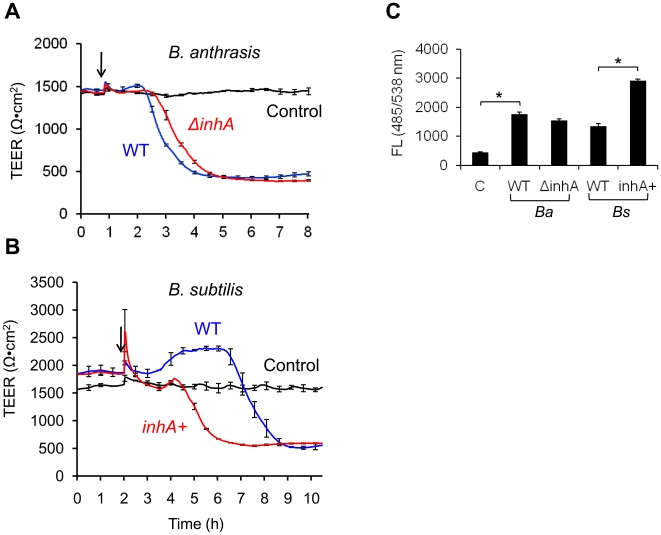
InhA contributes to increased permeability of infected HBMEC monolayers. (A, B) Real-time TEER of HBMECs (about 6×10^4^ cells/well) treated with *ΔinhA* of *B. anthracis* Ames 35 (*ΔinhA*, red line in panel A) and InhA-expressing *B. subtilis* (*inhA+*, red line in panel B) at MOI of 10. Signals corresponding to parental strains (WT) and culture medium without bacteria are shown as blue and black lines, respectively. Arrows indicate time points when bacteria were added to the cells. (C) Transwell permeability assays of HBMECs infected with *B. anthracis* (*Ba*) and *B. subtilis* (*Bs*) strains as indicated above for 4 h. Leakage of FITC-dextran added to the monolayers for 2 h was determined by measuring fluorescence in the bottom chamber at 485/538 nm. *P<0.01.

The results presented above strongly suggest that InhA is a potent enzyme capable of increasing barrier permeability of HBMEC monolayers *in vitro*. To explore whether InhA can disrupt the integrity of HBMEC monolayers during the infectious process, we first conducted real-time TEER assays using HBMECs exposed to growing bacteria. Initial experiments with the *ΔinhA* of *B. anthracis* Ames 35 in comparison with the wildtype detected a reduced capacity of *ΔinhA* to disrupt the HBMEC barrier (Figure 3A). However, the *inhA* deletion had a relatively small effect on the dynamics of TEER drop, indicating that pathogenic factors other than InhA (e.g. toxins) also may contribute to the TEER changes. To study the effect of InhA in the absence of toxin expression, we generated InhA-expressing non-pathogenic *B. subtilis* [24]. *B. subtilis inhA* transformant induced approximately a 2 h earlier TEER drop than the wildtype *B. subtilis* 168 (Figure 3B). The results of TEER measurements are supported by the transmigration assay of infected monolayers using FITC-conjugated dextran (Figure 3C). Western blot analysis of bacterial cells and culture supernatants confirmed that *B. subtilis inhA*-transformant expressed InhA at the level comparable with *B. anthracis* Ames 35 (not shown). These data demonstrate that InhA produced by growing bacteria contributes to a combined permeabilizing effect of *B. anthracis* pathogenic factors on HBMECs monolayers.

**Figure 4:**
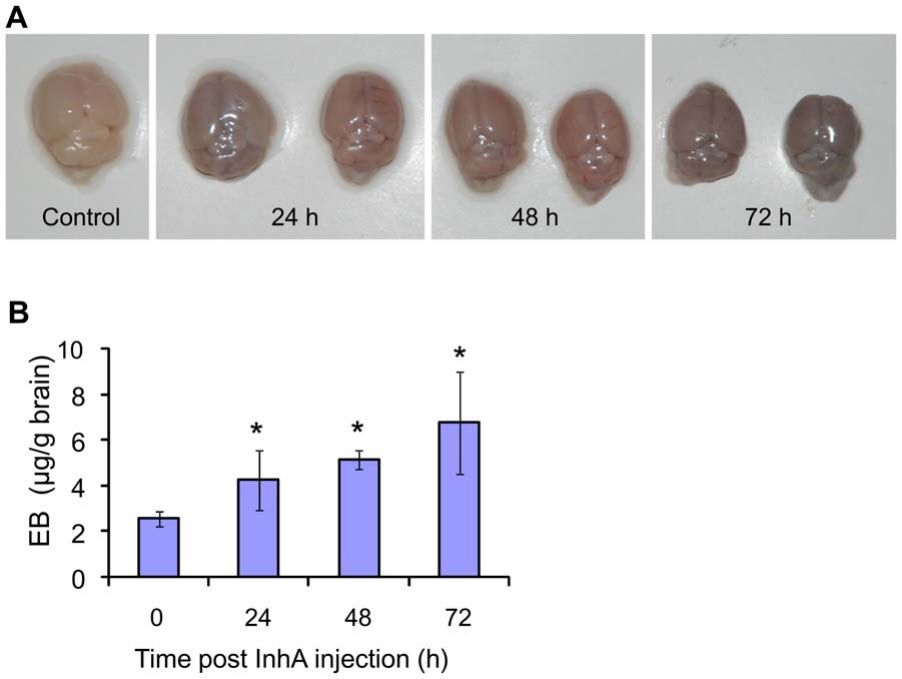
EB extravasation in the brains of InhA-challenged mice. Brain vascular permeability was measured using EB dye extravasation from whole mouse brains after challenge with InhA through retro-orbital injection. (A) Representative pictures of mouse brains at indicated time points. (B) Quantitation of the EB dye extravasation in the brain. The mean ± SD are presented as µg of EB dye per gram of brain (*n* = 5 in the InhA-injected groups; *n* = 3 in the control group). *P<0.05 *vs.* control group.

To extend this observation to *in vivo* BBB permeability, Evans Blue (EB) dye extravasation assay in the mouse brain was performed. Normally, the dye administered into circulation is not able to cross the BBB. However, dye can extravasate into the brain tissue if the BBB permeability is increased. Mice were injected with purified InhA and EB dye was injected i.v. approximately 1 h before euthanization. Digital images were taken immediately after the brain was excised from the body to visualize the amount of dye that penetrated into brain parenchyma (Figure 4A). The amount of extravasated EB dye extracted from the brain tissues was determined colorimetrically. Mice challenged with purified InhA had a significant 2-fold increase in dye leakage compared to control observed up to 72 h post challenge (Figure 4B).

**Figure 5:**
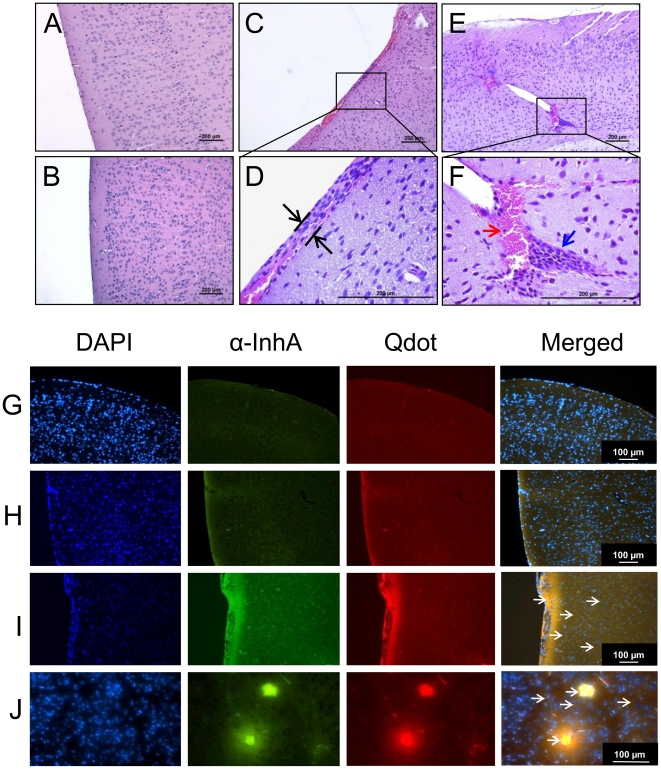
Mice administered nanobead-conjugated InhA demonstrate brain pathology associated with anthrax meningitis. Nanobeads conjugated to (active and heat-inactivated) InhA (1 U in 100 µl) were injected *via* retro-orbital route (*n* = 5). After 24 h, animals were euthanized and brains were harvested, fixed, and 5-µm sections were stained with hematoxylin and eosin (H&E) (A–F) or immunostained for InhA (G–J). Representative images are shown. (A) Brain of a mouse injected with unconjugated nanobeads. (B) Brain of a mouse injected with nanobeads conjugated to heat-inactivated InhA (C, E) Brain of mouse injected with nanobeads conjugated to active InhA, showing hemorrhagic areas. (D, F) Enlargements of marked areas in C and E, respectively. Meningeal thickening, hemorrhages and leukocyte infiltration are shown by double, red, and blue arrows, respectively. (G–J) Immunofluorescent visualization of nanobeads (red) and InhA (green). (G) Brain meninges from a mouse injected with nanobeads alone. (H) Brain meninges from a mouse injected with heat-inactivated InhA-conjugated beads. (I) Brain meninges from a mouse injected with active InhA-conjugated nanobeads. (J) Brain parenchyma of a mouse injected with active InhA-conjugated nanobeads. Infiltration of InhA-conjugated nanobeads in meninges and parenchyma is visible as numerous dots and areas of yellow color (overlapping red and green) indicated by arrows. Scale bar, 100 µm. DAPI was used to visualize cell nuclei.

Histological approaches were next used to examine correlation of the observed InhA effect with anthrax CNS pathology. Mice were injected with fluorescent nanoparticles conjugated with purified InhA. Fluorescence microscopy was used to examine the effect of InhA-conjugated nanoparticles on the BBB, as well as to visualize InhA localization within the mouse brain. Histological analysis of brain tissues showed that mice treated with either unconjugated nanoparticles (Figure 5A) or heat-inactivated InhA-conjugated nanoparticles (Figure 5B) did not exhibit any pathological change thereby eliminating nonspecific effects of nanoparticles or trauma of the brain due to injection. On the other hand, mice injected with nanoparticle-conjugated to InhA showed considerable leptomeningeal hemorrhaging, thickening, and infiltration of inflammatory cells (Figures 5C, D). These mice also displayed hemorrhages of the brain parenchyma (Figures 5E, F). Immunofluorescence analysis of similar sections indicated accumulation of active InhA-conjugated nanoparticles in areas corresponding to meningeal thickening and hemorrhage in brain microvasculatures (Figure 5I, J). No prominent nanoparticle fluorescence was detected in the brains of mice treated with nanoparticles alone or in mice treated with the heat-inactivated InhA-conjugated nanoparticles (Figure 5G, H). These results show that the nanoparticle-conjugated InhA is able to cross the BBB, gain access to the CNS parenchyma and induce anthrax meningitis-associated brain pathology due to activity of InhA [26].

**Figure 6:**
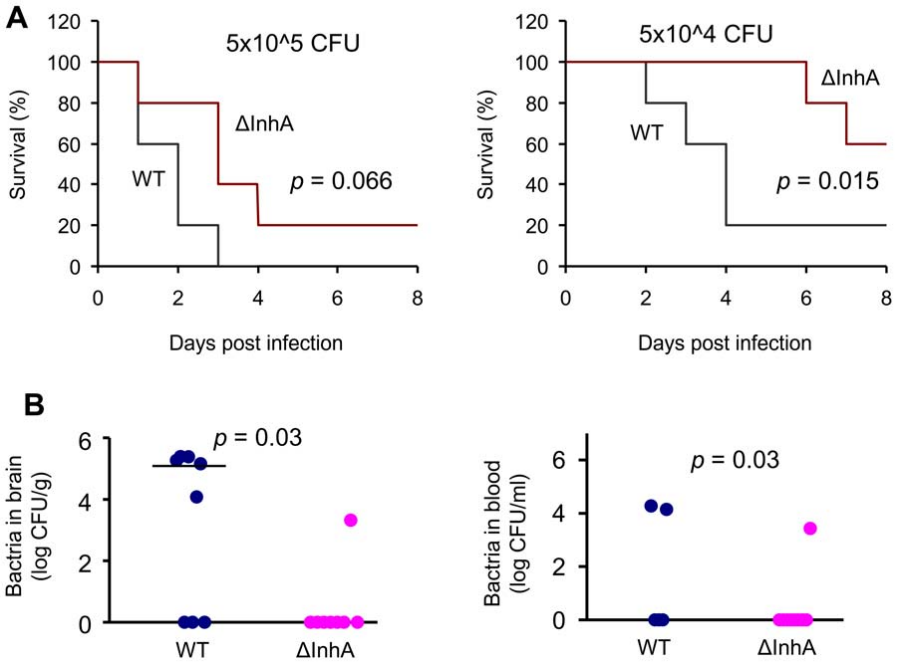
InhA contributes to virulence of *B. anthracis* infection in mice. Animals were injected intravenously into the tail vein with indicated CFU of the early-log-phase vegetative bacteria of Ames 35 (WT) and *ΔinhA* strains, correspondingly. Mice were monitored twice daily for mortality and survival. (A) Kaplan–Meier survival curve (*n* = 5 in both groups). Control mice (*n* = 3) injected intravenously with PBS did not die (data not shown). Data were analyzed using the log-rank test. (B) Bacterial load in brains (left panel, *n* = 8) and blood (*n* = 5, right panel) in mice challenged with 5×10^5^ CFU as in (A) at 48 h post challenge. Bar in (B) represents median bacterial CFU.

### InhA mutant bacteria display an attenuated response in mice

To demonstrate contribution of InhA to anthrax meningitis *in vivo*, mice were injected intravenously *via* the tail vein with early-log-phase vegetative bacteria of wildtype and *ΔinhA B. anthracis* Ames 35 strains. The intravenous route of infection and the choice of vegetative cells, as opposed to the commonly used challenge with spores, were chosen to model the late stage of the infectious process when systemic bacterial dissemination to the brain may lead to meningitis.

**Figure 7:**
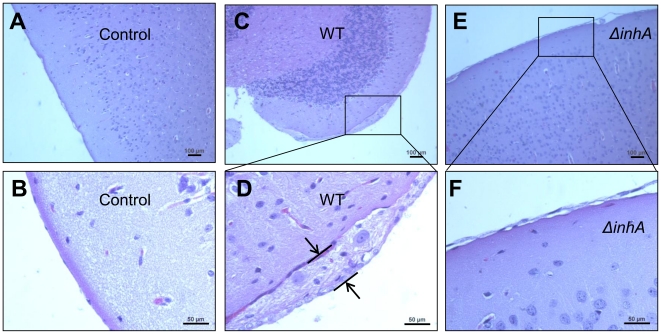
InhA contributes to BBB breakdown during *B. anthracis* infection in mice. Histopathology of representative H&E-stained brain sections from mice challenge as in Figure 6. (A, B) Control mice; (C, D) Moribund mice challenged with *B. anthracis* Ames 35. Meningeal thickening is shown by double arrows. (E, F) Moribund mice infected with *B. anthracis ΔinhA*. Boxes in C, E show the areas enlarged in D, F, respectively.

Mortality curves showed that the *ΔinhA* strain exhibited a prolonged mean time-to-death compared to the wildtype suggesting that InhA contributes to *B. anthracis* virulence (Figure 6A). The effect of *InhA* deletion was most reliable (*p* = 0.015) at the dose providing 80% mortality after challenge with the parental strain, when animals were not overwhelmed with a high dose of pathogen. Blood and brains obtained from moribund animals at 48–72 h post infection were analyzed for bacterial load. Five out of eight WT-infected mice had high bacterial counts in the brain, while in the case of *ΔinhA*-infected mice only one animal demonstrated presence of bacteria in the brain (Figure 6B). Upon microscopic examination of brain tissues, five out of six WT-infected mice (84%) showed meningeal thickening, compared to only one out of five *ΔinhA*-infected mice (20%). In moribund mice, WT infection (Figure 7A, D) showed more severe features of meningeal thickening compared to *ΔinhA* infection (Figure 7E, F). Collectively, these data demonstrate that InhA is involved in breaching the BBB and hemorrhagic brain damage contributing to the development of meningitis and *B. anthracis* virulence.

## Discussion

InhA is a secreted pathogenic factor of closely related Gram-positive bacteria, *B. thuringiensis*, *B. cereus* and *B*. *anthracis* [24]. It contributes to pathogenicity through several mechanisms, including the cleavage of antibacterial proteins, escape of bacteria from macrophages, control of blood coagulation, and degradation of matrix-associated proteins [12], [13], [24], [27]–[28]. However, a possible role for InhA in development of anthrax meningitis has not been demonstrated. We undertook this investigation to get insight into potential molecular targets of InhA in the BBB during disseminated *B. anthracis* infection. We suggest a novel mechanism of BBB breaching which involves InhA mediated proteolytic cleavage of the TJ protein ZO-1. To date, no other CNS pathogen is known to use this process to cross the BBB.

Our conclusion is supported by several lines of evidence. We first demonstrated that purified InhA caused a marked drop in TEER and morphological changes in cultured HBMECs which were absent in the case of another major *B. anthracis* protease, Npr599. These data indicate that InhA specifically targets the BBB protein(s) responsible for permeability of HBMEC monolayers *in vitro*. Previous studies have established that the barrier function of BBB can be attributed to the TJ proteins of endothelial cells. Alteration of TJ takes place in many CNS diseases, particularly in meningitis [15]. Our analysis of major TJ proteins revealed a unique susceptibility of ZO-1, but not occludin, claudin-1 or JAM-1, to InhA proteolytic degradation. Experiments with HBMECs pre-treated with cytochalasin D indicate that degradation of the intracellular ZO-1 protein by exogenous InhA requires an endocytic process that involves actin polymerization.

Our TEER measurements of HBMEC monolayers exposed to growing bacteria are also consistent with the suggested role of InhA in breaching the BBB. However, interpretation of these results is complicated by the fact that *B. anthracis* produces a number of pathogenic factors capable of compromising barrier function of epithelial and endothelial cells. In addition to LT and ET [29], [30], these factors include proteolytic and hemolytic enzymes as well as the pore-forming toxin, anthrolysin O [6], [31]. These difficulties were mainly avoided using the recombinant, non-pathogenic *B. subtilis* secreting InhA into culture medium. As expected, this strain induced a substantial acceleration of TEER drop in comparison with wildtype *B. subtilis* 168.

The next line of evidence includes the effect of purified InhA administration on BBB integrity *in vivo*. Histopathological examination of brain sections showed thickening of meninges and hemorrhage after treatment with InhA. These results are almost identical to the pathologies displayed in mice infected with *B. anthracis* [26]. In comparison with InhA, mice and rats treated with anthrax toxins develop widespread tissue disruption and vascular damage with focal hemorrhaging; however, histopathological lesions in brain meninges have not been reported [29], [30].

Finally, we used the murine model of disseminated anthrax where vegetative bacteria were injected intravenously to mimic the late stage of the disease. Previous studies of the *B. anthracis* secretome show that InhA is one of the abundant proteins expressed *in vivo* during *B. anthracis* infection [32]. Using *inhA* mutants of *B. anthracis* in comparison with wildtype we demonstrated that *inhA* deletion is associated with decreased virulence as well as lower bacterial loads in the blood and brain. Microscopic analysis of the brain sections confirmed that InhA is required for the development of meningitis. However, the effect of *inhA* deletion may not be limited just to the BBB permeability. Lower bacterial loads indicate that the overall less virulent mutant bacteria do not survive as well in the blood and therefore may not even reach the brain. On the other hand, InhA was previously implicated in the survival of germinating spores within host macrophages at the early stage of infection [24]. Therefore, in the comparative experiments with the *ΔinhA* mutant and wildtype strains aimed to reveal the late effect of InhA in the brain, it was important to avoid the contribution of the macrophage-related effect of InhA, which could potentially lead to misinterpretation of results due to increased killing of the *ΔinhA* mutant by macrophages.

Although the detailed mechanism of anthrax meningitis remains to be elucidated, available data allow us to suggest that the effect of InhA on the brain may have relevance to its capacity to induce thrombosis and blood coagulation [11]–[13], [28]. One of the key features found in clinical autopsies of patients dying of anthrax meningitis are massive hemorrhages, clotting, and swelling of the leptomeninges and intra-parenchymal regions of the brain associated with disseminated intravascular coagulation [33]–[35]. It can be surmised that once bacteria reach areas of the capillaries where they can no longer physically move, local secretion of InhA can quickly exceed the threshold level causing blood coagulation and local vascular damage [13], thus allowing the bacteria to spread to brain parenchyma. However, it seems certain that breaching of the protective barriers in the infected host represents a complex picture resulting from a combined activity of multiple pathogenic factors at different stages of disease. In addition to the InhA-mediated proteolytic damage, the results of ours and others indicate that mechanisms contributing to the BBB permeability in anthrax may include the host acute-phase response induced by InhA [28], the effects of LT on endothelial cells [36], [37], loss of extracellular syndecan [11], [35], [38], perturbation of cell-cell signaling in adherence junctions [39], and oxidative cell damage by anthrolysin O and bacterial fermentation products [31]. Biologically relevant concentrations of these factors at the sites of pathology and their relative contribution to mortality as well as their interplay with the major effects of LT and ET, need to be addressed in future studies.

In conclusion, we report here that InhA-induced BBB disruption provides a mechanistic model relevant to anthrax meningitis. We have identified a protease, its primary substrate, and its unique barrier permeability property in the closely structured human brain endothelia. The results of our *in vitro* experiments with HBMECs, as well as *in vivo* findings in mice lead us to conclude that InhA invades the endothelial cells, degrades the TJ protein ZO-1, and thus contributes to hemorrhage in the CNS. Taken together, the reported results add a new dimension to our understanding of hemorrhagic meningitis in *B. anthracis* infections. As InhA appears to be a potential pathogenic factor, it can be a molecular target for prevention of, and therapy against fatal hemorrhages associated with *B. anthracis* infection.
